# Supplementary material for: Inequalities in ownership and availability of home-based vaccination records in 82 low- and middle-income countries
Source: BMJ Glob Health. 2024 Dec 27;9(12):e016054. doi: 10.1136/bmjgh-2024-016054 (PMC11683979; doi:10.1136/bmjgh-2024-016054)
Supplement: online supplemental file 1 [file bmjgh-9-12-s001.pdf]

Supplementary materials

ST 1 – Surveys included in the analyses.

ISO code	Country	Year	Source
AFG	Afghanistan	2015	DHS
DZA	Algeria	2018	MICS
AGO	Angola	2015	DHS
ARM	Armenia	2015	DHS
BGD	Bangladesh	2017	DHS
BLZ	Belize	2015	MICS
BEN	Benin	2017	DHS
BIH	Bosnia and Herzegovina	2011	MICS
BFA	Burkina Faso	2010	DHS
BDI	Burundi	2016	DHS
CAF	CAR	2018	MICS
KHM	Cambodia	2014	DHS
CMR	Cameroon	2018	DHS
TCD	Chad	2014	DHS
COL	Colombia	2010	DHS
COM	Comoros	2012	DHS
COG	Congo Brazzaville	2014	MICS
COD	Congo Democratic Republic	2017	MICS
CRI	Costa Rica	2018	MICS
CIV	Cote dIvoire	2016	MICS
DOM	Dominican Republic	2019	MICS
EGY	Egypt	2014	DHS
SLV	El Salvador	2014	MICS
SWZ	Eswatini	2014	MICS
FJI	Fiji	2021	MICS
GAB	Gabon	2012	DHS
GMB	Gambia	2019	DHS
GHA	Ghana	2017	MICS
GTM	Guatemala	2014	DHS
GIN	Guinea	2018	DHS
GNB	Guinea Bissau	2018	MICS
GUY	Guyana	2019	MICS
HTI	Haiti	2016	DHS
HND	Honduras	2019	MICS
IND	India	2019	DHS
IDN	Indonesia	2017	DHS
IRQ	Iraq	2018	MICS
JAM	Jamaica	2011	MICS
JOR	Jordan	2017	DHS
KEN	Kenya	2014	DHS
KIR	Kiribati	2018	MICS
XKX	Kosovo	2019	MICS
LAO	Lao	2017	MICS

LSO	Lesotho	2018	MICS
LBR	Liberia	2019	DHS
MDG	Madagascar	2021	DHS
MWI	Malawi	2019	MICS
MDV	Maldives	2016	DHS
MLI	Mali	2018	DHS
MRT	Mauritania	2019	DHS
MEX	Mexico	2015	MICS
MNE	Montenegro	2013	MICS
MOZ	Mozambique	2015	DHS
MMR	Myanmar	2015	DHS
NAM	Namibia	2013	DHS
NPL	Nepal	2019	MICS
NER	Niger	2021	DHS
NGA	Nigeria	2018	DHS
PAK	Pakistan	2017	DHS
PNG	Papua New Guinea	2016	DHS
PRY	Paraguay	2016	MICS
PER	Peru	2020	DHS
PHL	Philippines	2017	DHS
RWA	Rwanda	2019	DHS
WSM	Samoa	2019	MICS
STP	Sao Tome and Principe	2019	MICS
SEN	Senegal	2019	DHS
SLE	Sierra Leone	2019	DHS
ZAF	South Africa	2016	DHS
PSE	State of Palestine	2019	MICS
TJK	Tajikistan	2017	DHS
TZA	Tanzania	2015	DHS
THA	Thailand	2019	MICS
TLS	Timor Leste	2016	DHS
TGO	Togo	2017	MICS
TUN	Tunisia	2018	MICS
TUR	Türkiye	2013	DHS
TUV	Tuvalu	2019	MICS
UGA	Uganda	2016	DHS
YEM	Yemen	2013	DHS
ZMB	Zambia	2018	DHS
ZWE	Zimbabwe	2019	MICS

ST 2 – Percentages of children aged 6-35 months according to home-based record ownership status stratified by age groups, wealth quintiles, mother's education, area of residence, antenatal care and institutional delivery

Group	Level	Card status	% Children	95% CI	
All children	6-35 months	Never had a card	10.2%	9.9%	10.5%
All children	6-35 months	Card seen	67.8%	67.4%	68.2%
All children	6-35 months	Has card, but not seen	12.8%	12.5%	13.0%
All children	6-35 months	No longer has a card	9.2%	9.0%	9.4%
Age group	6-11 months	Never had a card	9.5%	9.0%	9.9%
Age group	6-11 months	Card seen	76.3%	75.7%	76.9%
Age group	6-11 months	Has card, but not seen	9.1%	8.7%	9.5%
Age group	6-11 months	No longer has a card	5.2%	4.9%	5.5%
Age group	12-23 months	Never had a card	9.7%	9.3%	10.1%
Age group	12-23 months	Card seen	69.9%	69.4%	70.4%
Age group	12-23 months	Has card, but not seen	11.9%	11.6%	12.2%
Age group	12-23 months	No longer has a card	8.5%	8.2%	8.8%
Age group	24-35 months	Never had a card	11.1%	10.7%	11.5%
Age group	24-35 months	Card seen	61.5%	60.9%	62.0%
Age group	24-35 months	Has card, but not seen	15.5%	15.1%	15.8%
Age group	24-35 months	No longer has a card	12.0%	11.6%	12.3%
Sex	Male	Never had a card	10.0%	9.6%	10.3%
Sex	Female	Never had a card	10.5%	10.1%	10.8%
Sex	Male	Card seen	68.3%	67.8%	68.8%
Sex	Female	Card seen	67.3%	66.8%	67.8%
Sex	Male	Has card, but not seen	12.7%	12.4%	13.0%
Sex	Female	Has card, but not seen	12.8%	12.5%	13.2%
Sex	Male	No longer has a card	9.0%	8.7%	9.3%
Sex	Female	No longer has a card	9.4%	9.1%	9.7%
Wealth quintiles	Poorest	Never had a card	17.1%	16.3%	17.9%
Wealth quintiles	Second	Never had a card	12.4%	11.8%	13.0%
Wealth quintiles	Third	Never had a card	9.4%	8.8%	10.0%
Wealth quintiles	Fourth	Never had a card	6.1%	5.6%	6.6%
Wealth quintiles	Wealthiest	Never had a card	3.7%	3.4%	4.1%
Wealth quintiles	Poorest	Card seen	62.4%	61.6%	63.1%
Wealth quintiles	Second	Card seen	67.1%	66.4%	67.8%
Wealth quintiles	Third	Card seen	69.4%	68.7%	70.1%
Wealth quintiles	Fourth	Card seen	70.8%	70.0%	71.7%
Wealth quintiles	Wealthiest	Card seen	70.8%	70.0%	71.6%
Wealth quintiles	Poorest	Has card, but not seen	11.5%	11.1%	11.9%
Wealth quintiles	Second	Has card, but not seen	11.6%	11.2%	12.1%
Wealth quintiles	Third	Has card, but not seen	12.3%	11.8%	12.8%
Wealth quintiles	Fourth	Has card, but not seen	13.6%	13.0%	14.2%
Wealth quintiles	Wealthiest	Has card, but not seen	15.5%	14.9%	16.2%
Wealth quintiles	Poorest	No longer has a card	9.1%	8.6%	9.5%

Group	Level	Card status	% Children	95% CI	
Wealth quintiles	Second	No longer has a card	8.9%	8.5%	9.3%
Wealth quintiles	Third	No longer has a card	8.9%	8.5%	9.3%
Wealth quintiles	Fourth	No longer has a card	9.5%	9.0%	10.0%
Wealth quintiles	Wealthiest	No longer has a card	9.9%	9.4%	10.5%
Mother's education	None	Never had a card	23.0%	22.1%	24.0%
Mother's education	Primary	Never had a card	10.7%	10.2%	11.2%
Mother's education	Secondary+	Never had a card	4.3%	4.1%	4.6%
Mother's education	None	Card seen	55.7%	54.9%	56.6%
Mother's education	Primary	Card seen	67.2%	66.5%	67.9%
Mother's education	Secondary+	Card seen	73.4%	72.9%	73.9%
Mother's education	None	Has card, but not seen	9.5%	9.1%	9.9%
Mother's education	Primary	Has card, but not seen	12.1%	11.7%	12.5%
Mother's education	Secondary+	Has card, but not seen	14.5%	14.1%	14.8%
Mother's education	None	No longer has a card	11.7%	11.3%	12.2%
Mother's education	Primary	No longer has a card	10.0%	9.6%	10.4%
Mother's education	Secondary+	No longer has a card	7.8%	7.5%	8.1%
Area of residence	Urban	Never had a card	6.3%	5.9%	6.7%
Area of residence	Rural	Never had a card	12.4%	12.0%	12.9%
Area of residence	Urban	Card seen	68.3%	67.7%	69.0%
Area of residence	Rural	Card seen	67.5%	67.0%	68.0%
Area of residence	Urban	Has card, but not seen	15.2%	14.8%	15.7%
Area of residence	Rural	Has card, but not seen	11.3%	11.1%	11.6%
Area of residence	Urban	No longer has a card	10.1%	9.7%	10.5%
Area of residence	Rural	No longer has a card	8.7%	8.4%	9.0%
Antenatal care	4+ visits	Never had a card	4.7%	4.5%	5.0%
Antenatal care	<4 visits	Never had a card	17.3%	16.7%	17.9%
Antenatal care	4+ visits	Card seen	73.2%	72.8%	73.6%
Antenatal care	<4 visits	Card seen	63.1%	62.5%	63.8%
Antenatal care	4+ visits	Has card, but not seen	13.8%	13.5%	14.1%
Antenatal care	<4 visits	Has card, but not seen	10.6%	10.2%	10.9%
Antenatal care	4+ visits	No longer has a card	8.2%	8.0%	8.5%
Antenatal care	<4 visits	No longer has a card	9.0%	8.7%	9.4%
Institutional delivery	Institutional	Never had a card	4.6%	4.4%	4.8%
Institutional delivery	Noninstitutional	Never had a card	25.4%	24.6%	26.3%
Institutional delivery	Institutional	Card seen	74.1%	73.7%	74.4%
Institutional delivery	Noninstitutional	Card seen	51.2%	50.4%	52.0%
Institutional delivery	Institutional	Has card, but not seen	13.2%	12.9%	13.4%
Institutional delivery	Noninstitutional	Has card, but not seen	11.6%	11.2%	12.0%
Institutional delivery	Institutional	No longer has a card	8.2%	7.9%	8.4%
Institutional delivery	Noninstitutional	No longer has a card	11.8%	11.4%	12.3%
Age group	6-11 months	Never had a card	12.9%	9.8%	16.8%
Age group	6-11 months	Card seen	83.2%	79.5%	86.3%
Age group	6-11 months	Has card, but not seen	1.5%	1.0%	2.1%
Age group	6-11 months	No longer has a card	2.4%	1.3%	4.5%

Group	Level	Card status	% Children	95% CI	
Age group	12-23 months	Never had a card	12.1%	9.4%	15.4%
Age group	12-23 months	Card seen	83.8%	80.0%	87.1%
Age group	12-23 months	Has card, but not seen	2.0%	1.4%	2.8%
Age group	12-23 months	No longer has a card	2.1%	1.2%	3.4%
Age group	24-35 months	Never had a card	15.7%	12.6%	19.5%
Age group	24-35 months	Card seen	79.8%	75.6%	83.4%
Age group	24-35 months	Has card, but not seen	2.1%	1.6%	2.8%
Age group	24-35 months	No longer has a card	2.4%	1.5%	3.7%
All children	6-35 months	Never had a card	13.7%	11.5%	16.3%
All children	6-35 months	Card seen	82.1%	79.2%	84.6%
All children	6-35 months	Has card, but not seen	1.9%	1.6%	2.4%
All children	6-35 months	No longer has a card	2.3%	1.6%	3.2%
Sex	Male	Never had a card	12.6%	10.0%	15.7%
Sex	Female	Never had a card	14.9%	12.3%	18.0%
Sex	Male	Card seen	83.5%	80.2%	86.3%
Sex	Female	Card seen	80.6%	77.1%	83.7%
Sex	Male	Has card, but not seen	1.9%	1.5%	2.5%
Sex	Female	Has card, but not seen	1.9%	1.4%	2.6%
Sex	Male	No longer has a card	2.0%	1.2%	3.3%
Sex	Female	No longer has a card	2.5%	1.7%	3.7%
Wealth quintiles	Poorest	Never had a card	25.4%	21.2%	30.2%
Wealth quintiles	Second	Never had a card	14.9%	10.6%	20.5%
Wealth quintiles	Third	Never had a card	11.2%	8.0%	15.4%
Wealth quintiles	Fourth	Never had a card	9.4%	5.9%	14.5%
Wealth quintiles	Wealthiest	Never had a card	6.9%	4.1%	11.5%
Wealth quintiles	Poorest	Card seen	71.0%	65.6%	75.8%
Wealth quintiles	Second	Card seen	81.9%	76.6%	86.2%
Wealth quintiles	Third	Card seen	85.1%	80.6%	88.8%
Wealth quintiles	Fourth	Card seen	85.8%	81.2%	89.5%
Wealth quintiles	Wealthiest	Card seen	87.3%	81.6%	91.5%
Wealth quintiles	Poorest	Has card, but not seen	1.9%	1.2%	3.0%
Wealth quintiles	Second	Has card, but not seen	1.6%	1.0%	2.5%
Wealth quintiles	Third	Has card, but not seen	1.7%	1.0%	2.8%
Wealth quintiles	Fourth	Has card, but not seen	1.8%	1.2%	2.6%
Wealth quintiles	Wealthiest	Has card, but not seen	2.7%	1.9%	3.6%
Wealth quintiles	Poorest	No longer has a card	1.7%	0.9%	3.2%
Wealth quintiles	Second	No longer has a card	1.6%	0.9%	3.0%
Wealth quintiles	Third	No longer has a card	2.0%	1.1%	3.7%
Wealth quintiles	Fourth	No longer has a card	3.0%	1.6%	5.4%
Wealth quintiles	Wealthiest	No longer has a card	3.1%	1.8%	5.3%
Mother’s education	None	Never had a card	33.7%	28.2%	39.7%
Mother’s education	Primary	Never had a card	17.3%	13.2%	22.4%
Mother’s education	Secondary+	Never had a card	1.6%	1.0%	2.6%
Mother’s education	None	Card seen	62.4%	56.6%	67.9%

Group	Level	Card status	% Children	95% CI	
Mother’s education	Primary	Card seen	77.6%	71.7%	82.6%
Mother’s education	Secondary+	Card seen	94.4%	93.3%	95.3%
Mother’s education	None	Has card, but not seen	0.5%	0.2%	1.1%
Mother’s education	Primary	Has card, but not seen	0.8%	0.3%	2.2%
Mother’s education	Secondary+	Has card, but not seen	3.2%	2.6%	3.8%
Mother’s education	None	No longer has a card	3.4%	1.8%	6.3%
Mother’s education	Primary	No longer has a card	4.2%	2.6%	6.7%
Mother’s education	Secondary+	No longer has a card	0.8%	0.4%	1.6%
Area of residence	Urban	Never had a card	8.7%	5.5%	13.3%
Area of residence	Rural	Never had a card	16.3%	13.4%	19.7%
Area of residence	Urban	Card seen	85.4%	80.2%	89.4%
Area of residence	Rural	Card seen	80.4%	76.8%	83.6%
Area of residence	Urban	Has card, but not seen	3.2%	2.6%	4.0%
Area of residence	Rural	Has card, but not seen	1.3%	0.9%	1.8%
Area of residence	Urban	No longer has a card	2.8%	1.6%	4.9%
Area of residence	Rural	No longer has a card	2.0%	1.3%	3.0%
Antenatal care	4+ visits	Never had a card	4.1%	2.8%	5.9%
Antenatal care	<4 visits	Never had a card	32.1%	27.2%	37.4%
Antenatal care	4+ visits	Card seen	91.4%	88.9%	93.4%
Antenatal care	<4 visits	Card seen	64.5%	59.1%	69.5%
Antenatal care	4+ visits	Has card, but not seen	2.1%	1.5%	2.8%
Antenatal care	<4 visits	Has card, but not seen	0.7%	0.3%	1.4%
Antenatal care	4+ visits	No longer has a card	2.4%	1.5%	4.0%
Antenatal care	<4 visits	No longer has a card	2.8%	1.7%	4.6%
Institutional delivery	Institutional	Never had a card	5.8%	4.2%	7.9%
Institutional delivery	Noninstitutional	Never had a card	39.2%	33.8%	44.9%
Institutional delivery	Institutional	Card seen	90.4%	88.0%	92.3%
Institutional delivery	Noninstitutional	Card seen	56.0%	49.7%	62.2%
Institutional delivery	Institutional	Has card, but not seen	1.8%	1.4%	2.3%
Institutional delivery	Noninstitutional	Has card, but not seen	0.9%	0.4%	2.1%
Institutional delivery	Institutional	No longer has a card	2.1%	1.3%	3.4%
Institutional delivery	Noninstitutional	No longer has a card	3.9%	2.5%	6.0%

ST 3 – Immunization coverage among children aged 12-23 months according to home-based record ownership status.

Vaccine	HBR status	Coverage	95% CI	
BCG	Never had a card	33.0%	31.5%	34.6%
BCG	Card seen	97.5%	97.4%	97.7%
BCG	Has card, but not seen	87.6%	86.6%	88.5%
BCG	No longer has a card	89.8%	88.9%	90.6%
POLIO 3	Never had a card	22.2%	20.7%	23.9%
POLIO 3	Card seen	89.7%	89.4%	90.0%
POLIO 3	Has card, but not seen	45.8%	44.4%	47.2%
POLIO 3	No longer has a card	39.9%	38.0%	41.9%
DPT 3	Never had a card	15.0%	13.9%	16.2%
DPT 3	Card seen	90.5%	90.2%	90.8%
DPT 3	Has card, but not seen	63.4%	62.0%	64.7%
DPT 3	No longer has a card	57.6%	55.8%	59.4%
DPT 1	Never had a card	28.7%	27.2%	30.2%
DPT 1	Card seen	97.2%	97.0%	97.3%
DPT 1	Has card, but not seen	84.1%	83.1%	85.0%
DPT 1	No longer has a card	84.5%	83.3%	85.6%
MCV	Never had a card	26.3%	24.9%	27.7%
MCV	Card seen	80.3%	79.8%	80.7%
MCV	Has card, but not seen	73.6%	72.4%	74.8%
MCV	No longer has a card	72.7%	71.1%	74.1%
FULL	Never had a card	6.1%	5.5%	6.8%
FULL	Card seen	75.5%	75.1%	75.9%
FULL	Has card, but not seen	37.4%	36.1%	38.8%
FULL	No longer has a card	27.0%	25.3%	28.7%
DPT dropout ratio	Never had a card	47.6%	44.7%	50.6%
DPT dropout ratio	Card seen	6.9%	6.6%	7.1%
DPT dropout ratio	Has card, but not seen	24.7%	23.3%	26.0%
DPT dropout ratio	No longer has a card	31.9%	30.0%	33.8%

Legend: HBR – home-based record; BCG – Bacille Calmette-Guérin; DPT – diphtheria-pertussis-tetanus; MCV – measles containing vaccine; FULL – full immunization coverage (BCG + DPT3 + Polio3 + MCV)

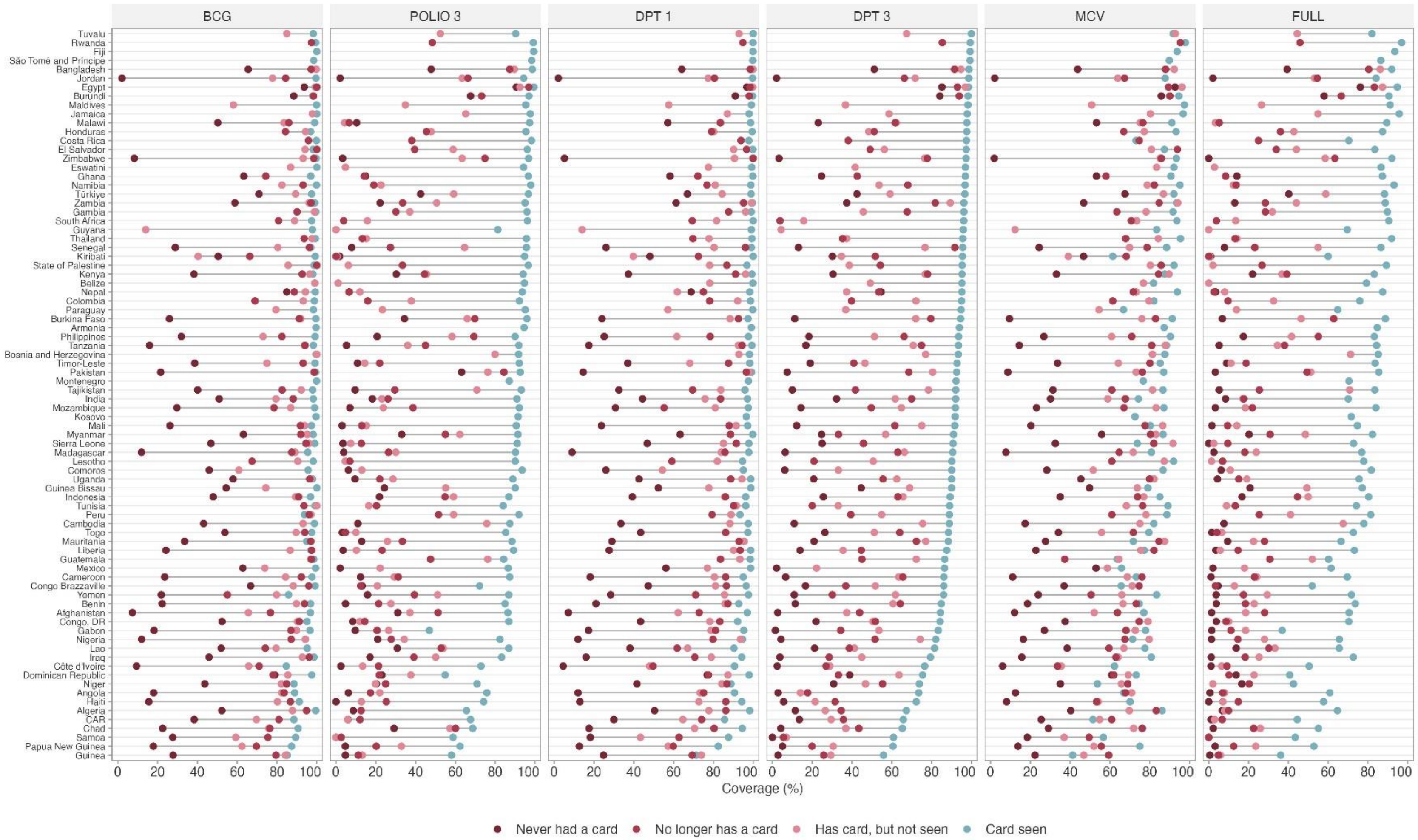

SF 1 – Immunization coverage among children aged 12-23 months according to home-based record ownership status by country. Countries ordered according to DPT3 coverage.
